# Editorial: New Advances in Cerebrovascular Disorders

**DOI:** 10.3390/jcm12185877

**Published:** 2023-09-10

**Authors:** Theodoros Karapanayiotides, Christos Krogias

**Affiliations:** 1 2nd Department of Neurology, Aristotle University of Thessaloniki, School of Medicine, AHEPA University Hospital, 54626 Thessaloniki, Greece; 2 Evangelisches Krankenhaus Herne, Department of Neurology, Academic Teaching Hospital, Ruhr University Bochum, 44801 Bochum, Germany; christos-krogias@ruhr-uni-bochum.de

Cerebrovascular disorders constitute major causes of disability and mortality worldwide. During the past decade, we have witnessed the development and establishment of mechanical thrombectomy (MT) as the new standard of care for acute stroke caused by emergent large vessel occlusion. Other advances in stroke management include the application of advanced neuroimaging to extend the time window of reperfusion treatments, optimal investigation of the subjacent pathologies of cryptogenic stroke, the widespread application of endovascular closure in patients with PFO-associated stroke, minimally invasive evacuation of intracerebral hematomas, and advances in the pathophysiology of stroke-related neuroinflammation and neurorepair. In this Special Issue, we focused on developing areas of clinical and basic stroke research. The studies which are presented highlight evolving diagnostic, treatment and prevention paradigms, as well as pathophysiological aspects of stroke, applying a multidisciplinary approach.

Carotid atheromatous disease accounts for a significant proportion of strokes in the anterior circulation. It is known that the degree of lumen stenosis is a major determinant of associated stroke risk, but is not the only one. Over the past 20 years and beyond, the interest of researchers has shifted from the carotid lumen-centered approach to the carotid wall-based approach in an attempt to identify “vulnerable” or high-risk atheromas [1]. Recently, this interest has been revived, as nonstenosing thrombogenic atheromas may be the underlying cause in the majority of the etiologically heterogeneous population of patients with embolic stroke of undetermined source [2]. Ultrasound, with its many advantages over other imaging modalities, holds a prominent position in the study of arterial wall pathology. In a comprehensive review, Alexandratou et al. summarize recent progress in the ultrasonographic assessment of vulnerable carotid atheromatic plaques and highlight the fields of future development in the evaluation of plaque echogenicity, of surface morphology, of the use of contrast-enhanced ultrasound and of elastography, in an attempt to communicate important take-home messages to clinicians [3]. In line with the previous review, Bill et al. investigated a large prospective cohort of stroke patients from the Lausanne stroke registry with carotid ultrasound within a week after onset [4]. They documented that compared with nondiabetics, diabetics—regardless of stroke etiology—have: (1) higher arterial resistance as quantified by the common carotid artery pulsatility index and (2) higher atherosclerotic burden as evidenced by the common carotid artery intima–media thickness. These ultrasound indexes were shown to be inter-related and also predictive of atheromatosis on CT angiography. The study highlights the importance of incorporating a dedicated neurosonology laboratory into a neurology department for the rapid identification of acute stroke patients with adverse vascular profile such as diabetics.

Despite its advantages, ultrasound remains a heavily operator-dependent modality. Therefore, its accuracy in documenting high-risk arterial wall pathology could be potentially improved by systemic blood biomarkers. Serum inflammatory biomarkers may represent surrogates for intraplaque inflammation; for instance, increased levels of C-reactive protein have been associated with plaque vulnerability [5]. Van Velzen et al. investigated whether white blood cells were associated with ≥50% internal carotid artery stenosis in acute stroke patients from the Preventive Antibiotics in Stroke Study [6]. Multivariate analysis showed that increased leukocytes (but not increased platelets) were independently associated with stenosis in men but not in women. In another prospective cohort of patients with acute stroke, Chondrogianni et al. investigated the association of omentin with patient clinical characteristics and outcomes [7]. Omentin is a novel fat-depot-specific adipokine and possesses anti-inflammatory, anti-diabetic and anti-atherogenic properties. In this study, increasing omentin levels were independently associated with higher stroke severity and with ipsilateral carotid stenosis. The above two studies underscore the need for future prospective research linking potential inflammatory biomarkers with imaging features of atheromas. Furthermore, patient outcomes should be evaluated along with ultrasound assessment of plaque echogenicity and with brain imaging to document infarct topography and extent.

MT is the latest milestone in vascular neurology and may be applied in populations where intravenous thrombolysis (iVT) is contraindicated or bears an increased bleeding risk, such as patients with malignancies. As randomized data are lacking, Aloizou et al. performed a systematic review of 18 relevant retrospective studies [8]. They concluded that in-hospital mortality, intracranial hemorrhage of any kind, reperfusion status and functional outcome upon discharge do not differ between patients with and without cancer. Conversely, 3-month functional outcomes and mortality seem to be worse in cancer patients. It remains unclear whether the latter is influenced by the endovascular intervention per se or by the progressing comorbidity of the underlying malignancy. Until then, it seems reasonable not to deny MT in patients with good pre-stroke functional status and a less aggressive or controlled neoplastic disease. One of the major determinants of functional outcome after stroke, and in particular after the application of reperfusion therapies, is the rate of development of stroke-associated pneumonia (SAP) during hospitalization. MT is more invasive than iVT and requires sedation or mechanical ventilation, which may increase the risk of SAP. To answer this question, Muhl et al. used data from a large prospective German stroke registry comparing patients undergoing reperfusion treatments for emergent large vessel occlusion of the anterior circulation [9]. Across the board, one out of five patients developed PAS and, as expected, SAP was associated with increased mortality and poor functional outcome; however, the rates of SAP between MT and iVT populations were not different. The authors concluded that in this unselected stroke population, MT with or without associated iVT did not influence the rate of SAP compared with patients undergoing exclusively iVT.

Beyond in-hospital and short-term complications of stroke, a major but often neglected source of post-stroke disability is the occurrence of depression (PSD). Selective serotonin reuptake inhibitors (SSRIs) are classically used to treat PSD and theoretically, their early initiation may confer “protection” against the development of PSD. To answer this important issue, Richter et al. performed a systematic review and meta-analysis [10]. They elegantly showed that early SSRI treatment reduced the relative risk of PSD by 25% compared to placebo at a cost of doubling the risk of bone fracture. Future studies should define high-risk candidates for PSD to improve the risk–benefit ratio of early post-stroke SSRI initiation.

The neurologic manifestations of systemic disease are diverse and often neglected unless meticulously sought for with the proper diagnostic modalities. The paper by Lubas et al. is a brilliant example of multidisciplinary approach with important clinical implications for patients suffering from ANCA-associated vasculitis with renal involvement (AAVR) [11]. The authors used noncontrast MRI sequences to detect brain parenchymal and small vessel involvement by the systemic vasculitis. It is remarkable that although CNS involvement on clinical grounds was found in 26% of the study population, MRI disclosed CNS vasculitis in 42%. This is remarkable, considering that MRI contrast sequences were not used in this renally impaired population, and offers a minimally invasive method for the follow-up of disease activity and control.

Cerebrovascular disorders are dominated by arterial diseases, with cerebral venous thrombosis (CVT) often being the “neglected child” of vascular neurology. However, in this Special Issue, Chen et al. describe a large retrospective cohort of Asian patients with CVT, evaluating clinical patterns of presentation, treatment paradigms and predictors of functional outcome [12]. They confirm that CVT in more than 60% of patients has a favorable outcome and that poor prognostic factors include disturbance of consciousness and high levels of D-dimers upon admission.

Since the introduction of iVT in 1995, stroke neurology has become a high-speed train uniting many medical specialties and striving to decrease the predominant cause of disability in the Western world. We hope that our Special Issue adds somewhat to the efforts of physicians and scientists fighting stroke worldwide but, most of all, we hope that it served as a query- and debate-generating standpoint because this is the essence of medicine. We thank the authors for their work, the reviewers for their valuable contributions and the *JCM* editorial team for their unrestrained support.

## Data Availability

Not applicable.
